# Tissue engineering strategies hold promise for the repair of articular cartilage injury

**DOI:** 10.1186/s12938-024-01260-w

**Published:** 2024-09-11

**Authors:** Chenhui Yang, Rongjin Chen, Changshun Chen, Fei Yang, Hefang Xiao, Bin Geng, Yayi Xia

**Affiliations:** 1 Department of Orthopedics, Lanzhou University Second Hospital, No.82, Cuyingmen, Chengguan District, Lanzhou, 730000 Gansu China; 2 Orthopaedics Key Laboratory of Gansu Province, Lanzhou University Second Hospital, Lanzhou, 730000 China; 3 The Second School of Clinical Medical, Lanzhou University, Lanzhou, 730000 China; 4 Department of Orthopedic, Tianshui Hand and Foot Surgery Hospital, Tianshui, 741000 China

**Keywords:** Osteoarthritis, Cartilage injury repair, Scaffold for tissue engineering, Stem cells, Chondrocyte, Cytokines, Hydrogel

## Abstract

Articular cartilage damage and wear can result in cartilage degeneration, ultimately culminating in osteoarthritis. Current surgical interventions offer limited capacity for cartilage tissue regeneration and offer only temporary alleviation of symptoms. Tissue engineering strategies are increasingly recognized as promising modalities for cartilage restoration. Currently, various biological scaffolds utilizing tissue engineering materials are extensively employed in both fundamental and clinical investigations of cartilage repair. In order to optimize the cartilage repair ability of tissue engineering scaffolds, researchers not only optimize the structure and properties of scaffolds from the perspective of materials science and manufacturing technology to enhance their histocompatibility, but also adopt strategies such as loading cells, cytokines, and drugs to promote cartilage formation. This review provides an overview of contemporary tissue engineering strategies employed in cartilage repair, as well as a synthesis of existing preclinical and clinical research. Furthermore, the obstacles faced in the translation of tissue engineering strategies to clinical practice are discussed, offering valuable guidance for researchers seeking to address these challenges.

## Introduction

Osteoarthritis (OA) is the most common form of arthritis and is associated with pain and loss of joint function. It imposes a huge burden on individuals and the social economy [1]. OA is becoming more common as the population ages and obesity increases [2]. Although the cause of OA can also be idiopathic, the disease is usually characterized by degeneration of the articular cartilage due to wear and injury. The study showed that patients with underlying cartilage damage were 7.4 times more likely to develop OA than those without damage [3]. Articular cartilage, subchondral bone, synovium, ligaments, and muscles change as a result of OA [4]. OA was originally thought to be an age-related disease, but recent studies have shown that it is a dynamic change caused by an imbalance between repair and destruction of cartilage damage and should be considered a syndrome [5]. Additionally, elevated levels of inflammatory components, metabolic changes, cellular aging, and mechanical load are associated with OA [6]. These factors lead to erosion of the cartilage surface, which in turn leads to matrix degradation and release of pro-inflammatory mediators during chondrocyte repair.

Traditional treatments for OA include nonsurgical treatment and surgical treatment. Nonsurgical treatment mainly used non-steroidal anti-inflammatory drugs for symptomatic treatment. Surgical treatment is for advanced joint replacement. Unfortunately, these measures do not reduce morbidity in the early stages of the disease, nor do they prevent cartilage degeneration and promote cartilage regeneration [7]. Therefore, understanding the pathophysiological factors and mechanisms of OA can provide a new method for more effective prevention and treatment of OA. Articular cartilage is a high-density tissue with significant load-bearing and low friction properties, allowing smooth movement of sliding joints. However, articular cartilage is limited to self-regeneration and repair due to its non-vascular, neuropathic, and non-lymphogenous nature. Cartilage tissue regeneration engineering is a promising method for repairing cartilage defects. Tissue engineering strategy combines engineering principles with cells, cytokines and biological materials to repair and improve cartilage tissue and thus achieves the purpose of repairing damaged cartilage [8]. Tissue engineering is an attractive method for repairing damaged articular cartilage, and studies have shown that it can repair damaged cartilage and restore its physiological structure and function [9, 10].

In this review, we first summarize the published tissue engineering strategies according to the properties of materials and different methods of use. Additionally, the basic and clinical studies on the application of these strategies in repairing articular cartilage lesions were summarized. Finally, we discuss the current barriers to clinical translation of tissue engineering strategies to encourage researchers in the field to overcome these challenges.

## Structure and function of articular cartilage

The function, composition, and structure of articular cartilage should be thoroughly understood before repairing articular cartilage injury. Under normal physiological conditions, articular cartilage is smooth and elastic, which cushions stress well to keep the joint sliding. The physiological properties of articular cartilage decline with age, and once this degradation begins, recovery is usually slow. Besides, after the injury of articular cartilage, this cushioning effect will also be significantly reduced, and then the injury and degeneration of articular cartilage will be gradually aggravated [12].

Articular cartilage is a dense connective tissue composed mainly of chondrocytes and extracellular matrix (ECM). Articular cartilage has a poor ability to heal itself due to lack of blood supply [13]. The cellular component of cartilage tissue consists of immature cells and mature cells. Immature cells in cartilage are chondroblasts, which have the ability to proliferate and secrete ECM and further differentiate into mature cells, such as chondrocytes. ECM is composed of water, collagen, proteoglycan, and other components, which mainly form the tissue structure of articular cartilage. Furthermore, hyaluronic acid (HA) and HA-associated proteins secreted by cells are also found in the ECM [14]. Type II collagen forms most of the dry weight of mature cartilage, while other types of collagen exist in the form of links to hyaluronic acid and proteoglycan, accounting for about 10% of their total weight [15]. Articular cartilage has neither nerves nor blood vessels, and its nutrition is mainly provided by synovial fluid around the synovial layer of the articular capsule and by arterial branches. The collagen fibers are interspersed with chondrocytes, which maintain the normal metabolism of articular cartilage. The mechanical properties of articular cartilage mainly depend on the triple helical structure of cartilage collagen fibers, which hold the negatively charged proteoglycans of the ECM [16]. The hydrophilicity of proteoglycan leads to its participation in the anti-compression loading of cartilage, which is based on the compressive action of water [17]. The hardness of cartilage is caused by the globular protein domain of proteoglycan [18]. HA is a non-sulfated glycosaminoglycan that wraps around every chondrocyte and whose rheological properties provide great tensile strength to the cartilage tissue [19]. HA is mainly involved in cell growth and migration. Its physical and chemical properties provide a transient hydration environment and promote cell migration by assisting cell detachment [20].

**Fig. 1:**
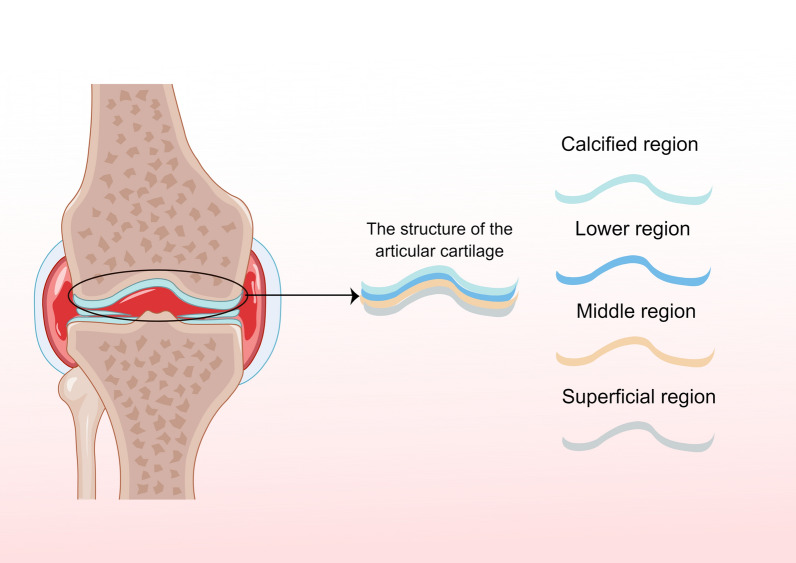
Articular cartilage structure is shown by Figdraw

Articular cartilage can be divided into four regions according to morphological characteristics (Fig. 1), which are calcified region, lower region, middle region and superficial region, and the cell distribution in these four regions is highly ordered [21]. These morphological features differ in genotype, phenotype and function [22]. A small amount of proteoglycan and collagen fibers were arranged in parallel in the superficial area, and chondrocytes were flat and elongated [23]. Moving to the middle area, the content of proteoglycan increases, collagen presents a disordered arrangement, and chondrocytes gradually become round [24]. Collagen fibers line the lower region perpendicular to the bone, and the main cell species are clustered in columns [25]. Closer to the calcified area, more chondrocytes tended to express multiple forms of collagen and ECM production increased [26]. Chondrocyte metabolism is affected by growth factors, electric field, matrix composition, mechanical load, and hydrostatic pressure. The generation of articular chondrocytes and different ECM molecules regulates the regeneration and remodeling of articular cartilage [27]. Therefore, it is particularly important to understand the components and structures of cartilage and the influencing factors of chondrocyte metabolism to optimize the strategies for promoting cartilage injury repair.

## Thermo-mechanobiology of cartilage

In articular cartilage, chondrocytes interact with their surrounding microenvironment through biological and physical signals to regulate cell behaviors such as cell proliferation and differentiation [28]. It has been shown that dynamic temporal interactions are mainly caused by the adhesion of chondrocytes to the extracellular matrix and the application of biomechanical stimulation. This interaction plays a crucial role in the transfer of force between cells, ultimately controlling chondrocyte function and tissue homeostasis, and can also be used to improve cartilage disease and directly promote regeneration [29]. To understand cartilage mechanobiology, it is crucial to understand how chondrocytes perceive physical signals, as well as how they translate these signals into biochemical signals [30].

The cartilage in the articular cartilage does not have the ability to regenerate, meaning that damage to the tissue cannot heal [31]. Meanwhile, traditional cartilage repair treatments do not achieve long-term function, rarely restoring the tissue to its original state, resulting in poor treatment outcomes [32]. Therefore, tissue engineering strategies are considered powerful tools for treating such injuries. Given the important role of biological and physical interactions between chondrocytes and their microenvironment in tissue formation and homeostasis, mechanobiology encourages the integration of various forms of stimulation into current cartilage engineering strategies [33]. Recently, Stampoultzis et al. [34] used a bioreactor that mimics joints to explore these interactions and revealed that dynamic thermomechanical stimulation enhances glycosaminoglycan and COL2a protein synthesis. These findings shed light on chondrocytes’ integration of various mechanobiological clues and provide valuable insights for improving extracellular matrix content in engineered cartilage construction. In addition, Stampoultzis et al. [35] also injected α-cyclodextrin and polyethylene glycol into the gelatin covalent matrix in the form of non-covalent polyurethane in order to enhance its kinetic properties. Subsequently, chondrocytes were implanted into these hydrogels and the effects of the supramolecular hydrogels on chondrocyte reactivity were systematically explored. The results revealed that supramolecular hydrogels enhanced the mechanical sensitivity of chondrocytes. In conclusion, the thermos-mechanobiology regulation of cartilage plays an important role in the repair of cartilage injury.

## Preclinical study of tissue engineering strategies applied to repair articular cartilage injury

### Simple biological scaffolds

**Fig. 2:**
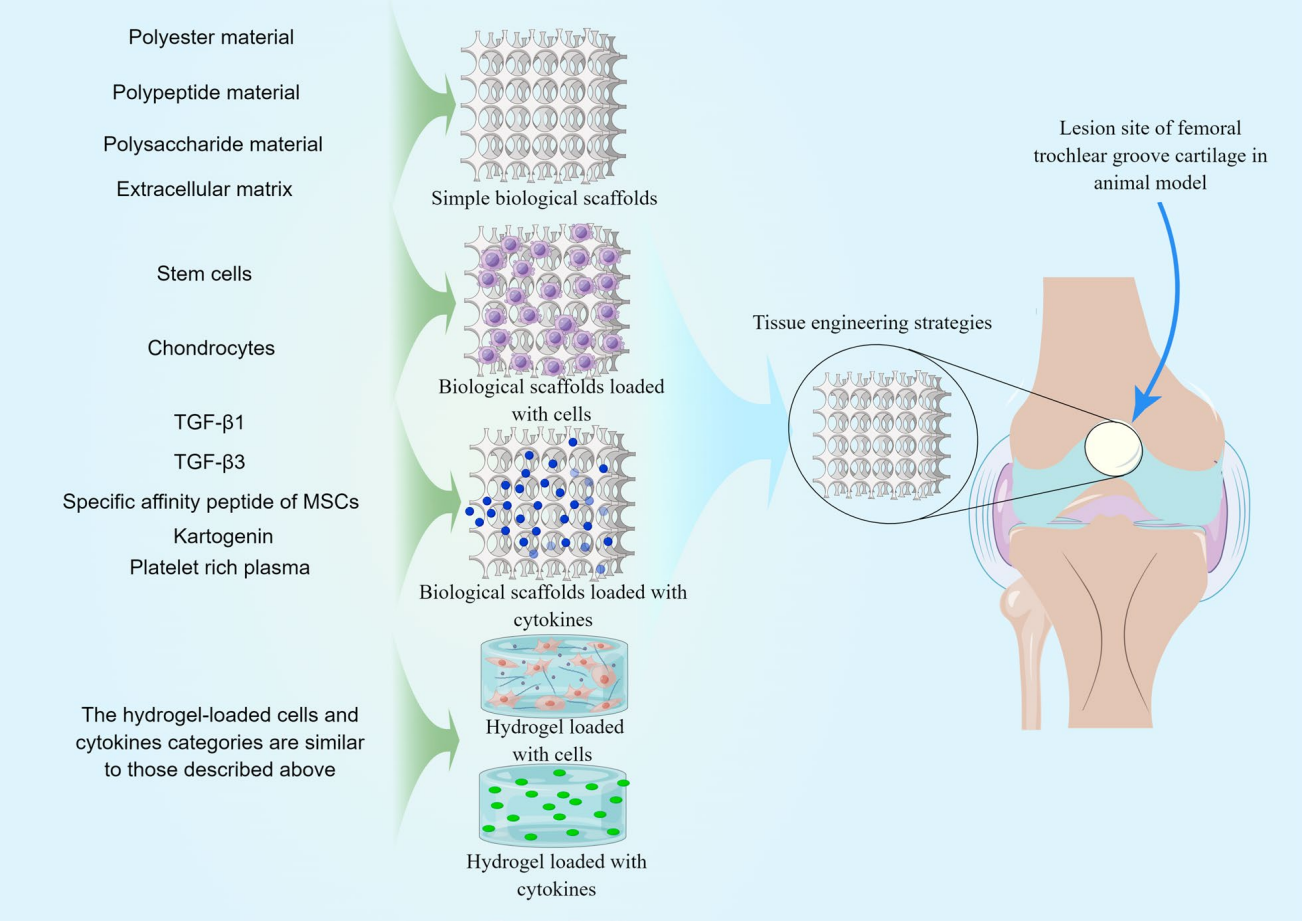
Summary of tissue engineering strategies for cartilage repair is presented by Figdraw

The ideal cartilage tissue engineering scaffold requires good biocompatibility, large pore structure, high mechanical strength and less trauma [36]. At present, the commonly used biological scaffolds are natural or synthetic polymer materials with high crystallization and high orientation. The most commonly used scaffolds for cartilage repair are polyester, polypeptides, polysaccharides and ECM materials (Fig. 2), which are mainly used to improve the mechanical properties and biocompatibility of scaffolds [37–39]. A large number of preclinical studies have been conducted using the above materials (Table 1**)** and good results have been achieved.

**Table 1:** Preclinical studies on the application of simple biological scaffolds in the repair of articular cartilage injury

Intervening measure	Joint site/implant site of cartilage injury models	Treatment outcomes	Animal model	References
Collagen scaffold	Trochlear groove of the femur	The collagen scaffold can promote the formation of hyaluronic cartilage repair, and its fibrous structure has no effect on the cartilage repair	Rabbits	De Mulder et al., [44]
Crosslinked HA-modified PCL scaffolds	Trochlear groove of the femur	The presence of HA improved the performance of scaffolds and showed good cartilage repair ability	Rabbits	Lebourg et al., [58]
PCL fiber membrane was prepared by electrospinning as periosteum scaffold	Trochlear groove of the femur	This scaffold was successfully used to regenerate osteochondral defects in rabbit models and the repair effect was greatly improved	Rabbits	Liu et al., [74]
Autologous BMSCs-derived ECM (aBMSCs-dECM) scaffold	Trochlear groove of the femur	aBMSCs-dECM scaffold enhance the therapeutic effect of articular cartilage repair	Rabbits	Tang et al., [72]
CS scaffolds enriched with D-raffinose	Trochlear groove of the femur and medial condyle of femur	In this study, CS scaffold rich in D-raffinose was found to be limited to cartilage regeneration	Rabbits	Ravanetti et al., [64]
A scaffold synthesized from copolymer of polyethyl acrylate and hydroxyethyl acrylate	Trochlear groove of the femur	This scaffold can guide the growth of cartilage tissue in vivo, suggesting the importance of stress transfer to cells in cartilage repair	Rabbits	Sancho-Tello et al., [9]
Type I collagen and hydroxyapatite composite scaffold	Trochlear groove of the femur	The composite scaffold fused well with the surrounding tissue and no signs of synovitis were observed	Pigs	Sosio et al., [47]
Three-dimensional porous SF scaffold prepared from non-mulberry silk	Subcutaneous	The scaffold showed biocompatibility 8 weeks after implantation in a subcutaneous rat model	Rats	Bhardwaj et al., [75]
PCL-PEG scaffold	Trochlear groove of the femur	This scaffold is a good cartilage tissue engineering scaffold, which can promote cell proliferation and adhesion, and has a good repair effect on cartilage defects	Rats	Fu et al., [40]
Tricalcium phosphate-collagen-HA (TCP-COL-HA) scaffold	Trochlear groove of the femur	TCP-Col-HA scaffold can be used as an effective carrier of cartilage regeneration cells	Rabbits	Meng et al., [59]
Human amniotic mesenchymal cells-derived ECM-PLGA scaffold	Trochlear groove of the femur	This kind of cell-free scaffold provides a good growth environment for MSCs and promotes the process of cartilage repair	Rats	Nogami et al., [73]
Collagen/SF composite scaffold incorporated with PLGA microsphere	Medial condyle of femur	This composite scaffold can promote the regeneration of articular cartilage and promote the fusion of the repaired cartilage with the surrounding cartilage	Rabbits	Wang et al., [48]
Parylene scaffold	Trochlear groove of the femur	Polyxylene scaffolds are a feasible choice for the treatment of cartilage injury, and the combined use of polyxylene scaffolds for superficial reconstruction surgery is better than that of single repair surgery	Rabbits	Franciozi et al., [76]
A cellular chondrocyte derived matrix and calcium phosphate matrix composite scaffold	Medial condyle of femur	Although this scaffold fused well with the recipient bone, the implant failed to produce a proper tissue repair effect	Horse	Vindas Bolaños et al., [69]
A biodegradable composite cartilage scaffold of PLGA, type I collagen and ceramic particles	Lateral condyle of femur	The scaffold achieved chondrogenesis within 6 months after implantation without adverse tissue reactions or other complications	Sheep	Yucekul et al., [10]
The composite scaffold of SF protein and chondroitin sulfate	Trochlear groove of the femur	The scaffold showed good anti-inflammatory effects both in vivo and in vitro, and promoted the repair of articular cartilage defects in animal models	Rabbits	Zhou et al., [51]
Three-dimensional collagen scaffold with shape memory properties	Subcutaneous	In this scaffold, chondrocytes have better proliferation, adhesion and redifferentiation abilities, which may contribute to improving the repair ability of cartilage	Rats	Jiang et al., [46]
PCL copolymer-based polyester polyurethane-urea (PCL-PU) scaffold	Medial condyle of femur	The PCL-PU **s**caffold could eventually be used in microfracture surgery to help repair cartilage	Rats	Shah et al., [41]
A decellularized cartilage matrix-derived scaffold	Trochlear groove of the femur	This scaffold can recruit endogenous stem cells into the site of cartilage injury and promote the differentiation of infiltrated cells into chondroblast cell lines	Rabbits	Sun et al., [67]
A bioactive porous resveratrol-polylactic acid-gelatin nanoscaffold	Condyles of femur	This scaffold promotes the repair of cartilage injury as a whole, and further studies have found the mechanism of accelerating cartilage repair through the PI3K/AKT signaling pathway	Rats	Yu et al., [43]
High hydrostatic pressure (HHP)-treated osteocartilage plugs	Trochlear groove of the femur and lateral condyle of femur	HHP-treated osteocartilage plugs can be used to fill knee osteocartilage defects and promote cell migration to the defect site	Rabbits	Hiemer et al., [65]
The composite nanofiber/ decellularized cartilage ECM (NF/DCECM) scaffold	Trochlear groove of the femur	NF/DCECM scaffold is promising tissue engineering scaffolds for cartilage regeneration and repair of cartilage defects	Rabbits	Li et al., [68]
An autologous BMSCs-derived ECM scaffold	Trochlear groove of the femur and medial condyle of femur	The use of ECM scaffold increased the number of BMSCs and improved the degree of cartilage repair	Rabbits and minipigs	Tang et al., [71]
A porous, CS/collagen-based scaffold	Trochlear groove of the femur	The scaffold has good mechanical resistance and no cytotoxicity, which is beneficial to tissue growth in vivo	Mice	Campos et al., [63]
A cell-free collagen I-based scaffold	Trochlear groove of the femur	Type I collagen scaffolds are highly biocompatible and can recruit host cells from surrounding joint tissues to facilitate cartilage repair of joint defects	Rats	Szychlinska et al., [45]
Cell-free biomimetic scaffold with cartilage ECM-like architectures	Trochlear groove of the femur	This scaffold can promote endogenous osteochondral regeneration	Rabbits	Zhang et al., [52]
A scaffold binding SF with elastin like polypeptide	Subcutaneous implantation	The scaffold mimics a three-dimensional cellular microenvironment to promote bone and cartilage regeneration	Nude mice	Chen et al., [53]
Two collagen (Col) scaffolds functionalized with a porcine decellularized ECM (dECM) in the forms of particle and solution named pE-Col and sE-Col	Trochlear groove of the femur	Compared with pE-Col scaffold, sE-Col scaffold achieved better structural hyaline neochondrogenesis and subchondral bone repair	Rabbits	Lu et al., [77]
A biomaterial combining recombinant human type III collagen (rhCo) and PLA	Medial condyle of femur	There was no difference between rhCo-PLA scaffold and spontaneous healing of cartilage injury	Pigs	Salonius et al., [50]
A PLGA scaffold filling a reactive oxygen species-scavenging hydrogel	Trochlear groove of the femur	Compared with PLGA scaffold, there was more deposition of glycosaminoglycans and type II collagen in the new cartilage in the mixed scaffolds, and they fused well with the surrounding tissues	Rabbits	Wu et al., [10]
A 3D-printed titanium alloy scaffold	Femoral trochlear groove	This scaffold promotes cartilage regeneration by providing mechanical support	Rabbits	Yang et al., [49]
Graphene-containing PCL scaffold	Femoral trochlear groove	PCL scaffold containing graphene significantly promoted the healing of large osteochondral defects	Rabbits	Basal et al., [42]
Bilayered ECM-derived scaffold	Femoral trochlear groove	The structure and composition of this scaffold can be spatially adjusted to guide the regeneration of osteochondral units	Caprine	Browe et al., [66]

HA: hyaluronic acid; PCL: poly ε-caprolactone; SF: silk fibroin; BMSCs: bone marrow mesenchymal stem cells; ECM: extracellular matrix; CS: chitosan; PEG: polyethylene glycol; PLGA: polylactic acid-glycolic acid; PLA: poly-lactide

#### Polyester material

Due to its mechanical properties, polyester materials are widely used in cartilage injury repair, such as polylactic acid (PLA), polyglycolic acid (PGA), polylactic acid-glycolic acid (PLGA), polyethyl acrylate and polycaprolactone (PCL). The poly-ε-caprolactone-polyethylene glycol (PCL-PEG) scaffold, as a biomaterial implant, has excellent cell adhesion, migration and proliferation performance in vitro. In vivo experiments, PCL-PEG membranes also showed good effect in promoting cartilage healing [40]. Similarly, Shah et al. [41] investigated whether PCL copolymer-based polyester urea (PCL-PU) scaffold contribute to the repair of cartilage defects and microfractures in vivo rat models. PCL-PU scaffold was significantly better than the control group in terms of cartilage defect filling and filling tissue characterization. PCL-PU scaffold can be used in conjunction with microfracture surgery to help repair cartilage. Recently, it has been found that different biomaterial properties of graphene combined with 3D-printed scaffolds produced by tissue engineering can be used for cartilage repair. Basal et al. [42] developed a graphene porous oriented PCL scaffold for the regeneration of large osteochondral defects. This scaffold was implanted in a rabbit model of full-layer osteochondral defect to evaluate the regeneration of the defect in vivo. The results showed that the improvement effect of graphene increased with increasing concentration. PCL scaffolds containing graphene significantly promoted the healing of large osteochondral defects.

Furthermore, polyethyl acrylate-hydroxyethyl acrylate copolymer scaffolds were constructed to study in rabbit cartilage repair models [9]. The study found that one week after implantation, the new tissue first grew in the deepest hole of the scaffold and then spread. After 3 months, the scaffold pore was dominated by cartilage tissue, and the bone tissue was near the subchondral bone. The study confirmed that this scaffold can guide the growth of cartilage tissue in vivo, suggesting the importance of stress transfer to cells in cartilage repair. In order to optimize the function of the polyester scaffold, Wu et al. [10] designed and prepared a new mixed scaffold by filling the hydrogel with active oxygen scavenging into the PLGA scaffold. In this study, the mixed scaffold was found to significantly regulate inflammatory response and promote hyaluronic cartilage regeneration after 12 weeks of implantation of whole-layer cartilage defects in rabbits. Compared with PLGA scaffolds, there was more deposition of glycosaminoglycans and type II collagen in the new cartilage in the mixed scaffolds group, and they fused well with the surrounding tissues. This study demonstrates the importance of polyester in conjunction with other pathways of action in promoting cartilage repair. Similarly, Yucekul et al. [10] developed a biodegradable composite cartilage scaffold of PLGA, type I collagen and ceramic particles for the repair of cartilage defects. Analysis of the study specimens showed that the implants achieved chondrogenesis within 6 months with no adverse tissue reactions or other complications reported. The results of this study suggest that porous biocompatible implants appear to be a promising treatment option for cartilage repair. Drug-loaded tissue engineering scaffolds of polyester materials also provide a feasible treatment option for the repair of cartilage injury. Yu et al. [43] designed bioactive porous resveratrol-PLA-gelatin nanoscaffolds for the repair of articular cartilage defects, and discussed the possible mechanism of successful repair. These results confirm that this scaffold promotes cartilage repair as a whole and explain the mechanism by which it may accelerate cartilage repair through the PI3K/AKT signaling pathway.

#### Collagen material

To induce hyaluronic cartilage repair, the investigators attempted cartilage repair with type I collagen scaffolds. De Mulder et al. [44] prepared type I collagen scaffold for cartilage repair. The results showed that collagen scaffold can promote the formation of hyaluronic cartilage repair in rabbit knee cartilage defects. Similarly, Szychlinska et al. [45] evaluated the ability of cell-free collagen I scaffold to promote cartilage repair after in situ implantation in vivo. An articular cartilage injury model was established in the trochlear groove of the femur of rats, and then type I collagen scaffold was implanted into the articular cartilage defects. The results indicate that type I collagen scaffold is highly biocompatible and can recruit host cells from the surrounding joint tissues to promote cartilage repair of joint defects, suggesting that type I collagen scaffold can be used as a potential method for cartilage tissue regeneration. Besides, to further enhance the ability of collagen scaffolds in cartilage repair, the researchers also attempted to optimize the structure by modifying the collagen structure. Jiang et al. [46] studied the advantages of collagen with triple helix structure in collagen-based cartilage engineering composites. The results showed that chondrocyte proliferation, adhesion and redifferentiation in collagen scaffolds with triple helix structure were better, which may contribute to the enhancement of cartilage repair ability. These studies suggest that collagen is a promising material for promoting cartilage injury repair, and researchers can improve its function in cartilage injury repair by optimizing its structure.

Researchers are also trying to use collagen in combination with other biomaterials to optimize its function in repairing cartilage damage. Sosio et al. [47] combined with type I collagen and hydroxyapatite to construct a three-dimensional (3D) scaffold for the repair of osteochondral injury in pig models. The results showed that the scaffold was well integrated with the surrounding tissue and no signs of synovitis were observed. It was also found that the quality of the repaired tissue appeared to be superior for the lesions treated with chondrocyte free scaffold, suggesting that type I collagen scaffold have a promising application in osteochondral repair surgery. Wang et al. [48] prepared a PLGA microsphere and collagen/silk fibroin (SF) composite scaffold that could be used for cartilage repair. The prepared composite scaffold was implanted into the rabbit total thick articular cartilage defect. The results showed that the collagen/SF composite scaffold could promote the regeneration of articular cartilage, and promote the fusion of repaired cartilage and surrounding cartilage. Therefore, the composite scaffold will be a promising material for cartilage repair and regeneration. Besides, Yang et al. [49] attempted to enhance cartilage repair function by combining collagen with 3D-printed titanium alloy scaffold. In vitro results confirmed the biocompatibility of the scaffold material, while in vivo results showed that the mechanical support provided by the 3D-printed titanium alloy layer in the rabbit osteochondral defect model accelerated osteochondrogenesis and fusion with adjacent host tissue after 24 weeks, playing an important role in the long-term regeneration of cartilage. This study suggests that collagen combined with mechanical support provided by 3D-printed titanium alloys promotes cartilage regeneration by providing a collaborative mechanical support platform. The above studies suggest that the combination of collagen with other biomaterials is a promising strategy for promoting cartilage repair. However, other studies investigating the cartilage repair ability of recombinant human type III collagen (Rhco) and PLA biomaterials have found no difference between the use of Rhco-PLA scaffolds and spontaneous healing [50]. Therefore, the collagen scaffold strategy needs to be further optimized in promoting cartilage repair.

SF can be used in tissue repair engineering because of its biocompatibility and biodegradability. Meanwhile, SF is widely used in structural materials because of its excellent mechanical properties, long-lasting in vivo stability and low immunity. However, the rapid degradation of pure SF scaffolds poses a challenge for effective reconstruction of new tissue similar to natural articular cartilage. Chondroitin sulfate is a glycosaminoglycan found in natural cartilage ECMs and exhibits a number of useful biological properties, including anti-inflammatory activity. Zhou et al. [51] reported that the combined application of SF protein and chondroitin sulfate could cooperatively promote the repair of articular cartilage defects, and the effect of cartilage repair in vivo was evaluated using rabbit osteochondral defect model. International Cartilage Repair Association (ICRS) histological evaluation showed that the SF-chondroitin sulfate scaffold induced more new tissue formation and better structural recovery after 6 and 12 weeks of implantation than the silk scaffold. Besides, the study also confirmed that the scaffold showed a good anti-inflammatory effect both in vivo and in vitro, and promoted the repair of articular cartilage defects in animal models. Similarly, Zhang et al. [52] developed a scaffold that provides natural ECM-SF. It was found that ECM-SF scaffold with vertically aligned pore structure ratio was favorable for the growth of endogenous bone marrow mesenchymal stem cells (BMSCs) and could promote the regeneration of endogenous osteochondral cells when the scaffold was implanted into rabbit osteochondral defects. In addition, Chen et al. [53] designed a scaffold binding SF with elastin like polypeptide (ELP). The results showed that the fusion of SF and ELP enhanced the function of the scaffold. BMSCs and chondrocytes showed better diffusion and proliferation on SF-ELP scaffold. In vitro and in vivo experiments further demonstrated that the use of SF-ELP scaffold enhanced the formation of mature bone and cartilage tissue compared to bare SF scaffold. These studies indicate that SF has a certain value as a biomaterial in repairing cartilage injury.

#### Polysaccharide material

HA is a glycosaminoglycan that is ubiquitous in vertebrates, including humans, and is involved in a variety of biological processes, such as cell differentiation, embryonic development, inflammation, and wound healing [54]. It interacts with chondrocytes through CD44 receptor, and positively affects proliferation, ECM secretion, phenotypic regulation and other pathways, and has a chondroprotective effect, which can counteract the effects of oxidative stress on chondrocytes [55–57]. Therefore, HA combined with bioengineering materials can be used in cartilage repair.

In recent years, researchers have tried to apply HA combined with bioengineering materials in cartilage repair. Lebourg et al. [58] prepared a HA-modified PCL scaffold for cartilage injury repair. After the implantation of PCL/HA scaffold into rabbit cartilage defects, spontaneous healing reaction was found in subchondral bone bleeding. The presence of HA improved the performance of the scaffold and supported a good repair response through international cartilage repair score and immunohistological evaluation. Meng et al. [59] developed a novel tricalcium phosphate-collagen-HA (TCP-COL-HA) scaffold, which can be used as stem cells carrier to induce chondrogenesis and promote cartilage repair. In this study, the TCP-COL-HA scaffold showed strong cartilage regeneration and fusion with surrounding tissues in rabbit osteochondral defect repair model. These results indicated that the addition of HA promoted the ability of the scaffold to induce cartilage, suggesting that the TCP-COL-HA scaffold could be used as an effective carrier of cartilage regeneration cells.

Chitosan (CS) is a natural source of polysaccharides. Due to its biodegradable, biocompatible and non-toxic properties, CS has been widely used in biomedical fields such as tissue engineering and drug preparation [60]. Previous studies have shown that CS has antibacterial and anticoagulant properties, promotes wound healing, and improves immune function [61, 62]. Therefore, researchers tried to use CS to construct biological scaffolds for cartilage repair. CAMPOS Y et al. [63] reported a study on a porous CS/collagen-based scaffold as an implant for repairing damaged articular cartilage. The scaffold showed good mechanical resistance and no cytotoxicity, was conducive to tissue growth in vivo, and remained stable in mice for 35 days. The results show the clinical potential of this porous CS/collagen scaffold in cartilage tissue engineering. However, Ravanetti et al. [64] found that CS scaffolds loaded with D-raffinose had no significant promoting effect on cartilage repair of rabbit osteochondral defect models. It was found that the recovered implants were surrounded by fibrous capsules and contained obvious inflammatory infiltration, and no hyaluronic cartilage was formed in the defect. This study highlights the importance of CS scaffolds in conjunction with other strategies in promoting cartilage regeneration.

#### Extracellular matrix

Although there have been some clinical advances in cartilage repair, the repair of osteochondral defects remains a major challenge. The ideal scaffold for cartilage repair should mimic the natural ECM and exhibit excellent properties such as biocompatibility, suitable porosity, and good cell affinity. The cartilage matrix has a strong ability to induce cartilage and can be used as a scaffold for cartilage reconstruction. In recent years, researchers have explored the use of scaffolds made by cartilage ECM to repair cartilage injury through a large number of studies, and some achievements have been made in preclinical studies [65–69].

To investigate the role of ECM components in promoting cartilage repair, a study evaluated the feasibility of using high hydrostatic pressure (HHP) inactivated osteochondral tissue to repair osteochondral defects in rabbits [65]. The results showed that the osteochondral thrombus treated with HHP could be used to fill the osteochondral defect of knee joint and promote cell migration to the defect site. In order to reproduce the complex hierarchical structure of native articular cartilage, Browe et al. [66] created a double-layer ECM-derived scaffold. In vitro and in vivo experiments demonstrated that this scaffold could preferentially guide mesenchymal stem cells (MSCs) to differentiate into chondroblast lines, thus promoting the regeneration of cartilage interface. Additionally, other researchers have attempted to prepare composite scaffolds of acellular cartilage matrix (DCM) combined with other biomaterials and explored their role in cartilage injury repair. Sun et al. [67] combined DCM-derived scaffolds with functionalized nanofiber hydrogels to repair rabbit osteochondral defects. The results of in vivo experiments showed that the composite scaffold has good function of hyaluroid cartilage repair and successful subchondral bone reconstruction. It can recruit endogenous stem cells into the damaged cartilage site and promote the differentiation of infiltrating cells into chondroblast cell lines. The researchers also attempted to produce a scaffold composed of gelatin-PCL nanofibers and decellarized cartilage ECM (DCECM) [68]. The results showed that the DCECM component of the composite scaffold promoted the early maturation of cartilage lacunae and the secretion of collagen and glycosaminoglycan. However, Vindas Bolaños et al. [69] studied acellular chondroid-derived matrix composite scaffolds with calcium phosphate in the repair of osteochondral defects and found that implants failed to produce reasonable repair tissues. These studies suggest that ECM-derived scaffolds and their composite scaffolds are promising tissue engineering scaffolds for cartilage regeneration and cartilage defect repair, but more studies are needed to confirm their efficacy in promoting cartilage repair.

In recent years, it has been found that the ECM derived from bone marrow MSCs can play a very good therapeutic effect in promoting cartilage repair [70]. Tang et al. [71] investigated the role of ECM scaffold derived from autologous BMSCs in cartilage repair in two animal models. The results of this study found that the use of this ECM scaffold increased the number of bone MSCs and improved the degree of cartilage repair in two animal models of knee cartilage repair. Similarly, Tang et al. [72] evaluated the effectiveness of autologous bone marrow mesenchymal stem cells-derived ECM (aBMSC-dECM) scaffold for cartilage repair after implantation of bone marrow stimulation (BMS). The results showed that implantation of aBMSC-dECM scaffold could promote the differentiation of MSCs through BMS to enhance the therapeutic effect of articular cartilage repair. These results suggest that the aBMSC-dECM scaffold may be a successful candidate for novel cartilage tissue engineering. Furthermore, the ECM of human amniotic mesenchymal cells (HAMs) has a variety of biological activities. A novel HAM-derived ECM-coated PLGA (ECM-PLGA) scaffold has been developed to investigate its potential as a cell-free scaffold for cartilage repair [73]. The results showed that after the ECM-PLGA scaffold implantation of osteochondral defects in the knee of rats, the tissue was gradually regenerated, and the repair effect of hyaline cartilage was significantly better than that of blank control group. The above studies indicate that the mesenchymal ECM can provide a good growth environment for MSCs and promote the process of cartilage repair. Therefore, mesenchymal ECM may be a promising strategy for cartilage repair.

### Biological scaffolds loaded with cells

As a scaffold type for cartilage repair, cell-loaded biological scaffolds have been widely used in preclinical studies. Currently, the main loaded cells are stem cells (MSCs of various tissue origin) and chondrocytes (Fig. 2). On the one hand, biological scaffolds can provide growth space for the above cells and promote chondrogenesis. On the other hand, the researchers used gene editing to control the differentiation of stem cells into chondrocytes. A large number of preclinical studies have also confirmed that tissue engineering scaffolds loaded with cells have great potential in promoting cartilage repair (Table 2).

**Table 2:** Preclinical studies on the application of cell-loaded biological scaffolds in the repair of articular cartilage injury

Intervening measure	Joint site/implant site of cartilage injury models	Treatment outcomes	Animal model	References
A three-dimensional porous acellular scaffold derived from ECM of natural human cartilage	Trochlear groove of the femur	The cartilage repair tissue induced by this cartilage ECM scaffold is comparable to that of natural cartilage in terms of mechanical properties and biochemical composition	Rabbits	Kang et al., [86]
MSCs-loaded bilayer PLGA scaffold	Trochlear groove of the femur	MSCs incorporating into PLGA scaffold can enhance the regeneration and fusion ability of repaired cartilage and surrounding cartilage	Rabbits	Qi et al., [91]
Connective tissue growth factor-modified BMSCs-PLGA scaffold	Femoral trochlear groove	BMSCs-PLGA scaffold may be an alternative treatment for large osteochondral defects in high-load sites	Rabbits	Zhu et al., [80]
MSCs-seeded polyethylene-oxide-terephthalate/polybutylene-terephthalate (PEOT/PBT) scaffold	Medial condyle of femur	PEOT/PBT scaffold loaded with MSCs was superior to those without scaffolds in promoting hyaluronic cartilage formation, creating a repair environment for cartilage repair	Rabbits	Barron et al., [79]
A composite scaffold through gene editing to control the chondrogenesis of ADSCs by transfecting the recombinant fusion protein LAP-MMP-mTGF-β3	Subcutaneous	The scaffold can accelerate the differentiation of ADSCs into cartilage in vivo	Mice	Zheng et al., [87]
Addition of MSCs to autologous platelet-enhanced fibrin scaffold	Lateral condyle of femur	The addition of MSCs to scaffold did not promote cartilage repair but stimulated bone formation in partial cartilage defects	Horses	Goodrich et al., [82]
Allogenic chondrocytes with CS hydrogel (CS)-demineralized bone matrix (DBM) hybrid scaffold (CS/DBM)	Femoral trochlear groove	The CS/DBM scaffold loaded with allogeneic chondrocytes can successfully repair rabbit cartilage injury in a single operation	Rabbits	Man et al., [88]
A new biphasic scaffold (type I collagen/hydroxyapatite) previously loaded or not with concentrated bone marrow cells	Femoral trochlear groove	Biphasic scaffolds loaded with concentrated bone marrow can significantly promote the healing of cartilage injury	Rabbits	Hernigou et al., [92]
A bilayered poly PLGA scaffold	Femoral trochlear groove and condyles of femur	The implantation of chondrocytes in the double-layer scaffold can promote the regeneration and integration of osteochondrosis, thus achieving the repair of articular cartilage	Pigs	Lin et al., [89]
Stromal vascular fraction cells (SVFs) and ADSCs co-cultured with chondrocytes seeding on scaffolds	Femoral trochlear groove	The scaffold loaded with SVFs and chondrocytes showed more desirable outcomes for healing cartilage injury in vivo	Rabbits	Ba et al., [90]
A CS/SF scaffold loaded with C-type natriuretic peptide gene-modified BMSCs	Trochlear grooves of the distal femur	The scaffold can promote the formation of more cartilage matrix	Rats	Yang et al., [81]
Three-dimensional printed PCL-hydroxyapatite scaffold coated with UCB-MSCs	Femoral trochlear groove	PCL-hydroxyapatite scaffold loaded with UCB-MSCs promote the repair of articular cartilage	Rabbits	Zheng et al., [83]
Human umbilical cord MSCs in a gelatin honeycomb scaffold	Subcutaneous	The scaffold can promote human umbilical cord MSCs survival and chondrogenic differentiation to form hyaluronic cartilage	Mice	Chang et al., [85]
Hydrogel-hydroxyapatite-monomeric collagen type-I scaffold	Lateral femoral condyles	This scaffold have the potential to enhance osteocartilage repair under low frequency electromagnetic field treatment	Rabbits	Yan et al., [93]
Gelatin/hydroxyapatite composite scaffold loaded with human umbilical cord blood-derived MSCs (hUCB-MSCs)	Femoral trochlear groove	This scaffold loaded with hUCB-MSCs can effectively repair cartilage defects	Pigs	Huang et al., [84]
Manganese-loaded composite scaffold combined with chondrocytes	Femoral trochlear groove	This scaffold promotes type II collagen formation and cartilage repair	Rats	Wei et al., [94]

PCL: poly ε-caprolactone; MSCs: marrow mesenchymal stem cells; BMSCs: bone marrow mesenchymal stem cells; ADSCs: adipose-derived stem cells; UCB-MSCs: umbilical cord blood-derived MSCs; ECM: extracellular matrix; CS: chitosan; PLGA: polylactic acid-glycolic acid

#### Hematopoietic stem cells (HSCs)

HSCs have great potential in cartilage repair due to their pluridirectional differentiation. A systematic review of the literature on HSCs-based tissue engineering strategies in animal models of cartilage repair was conducted to identify trends and clarify the use of HSCs in cartilage repair [78]. The results of this study showed that the implant containing HSCs performed better on cartilage regeneration in animal models than the scaffold group alone. Umbilical cord MSCs and HA-containing scaffolds were popular stem cell and scaffold choices, respectively. This study highlights the potential of HSCs for cartilage regeneration in vivo and the importance of selecting appropriate biological scaffolds to assist in enhancing cartilage repair.

#### Marrow mesenchymal stem cells (MSCs)

Barron et al. [79] evaluated the effectiveness of polyethylene terephthalate/polybutyl terephthalate (PEOT/PBT) scaffolds loaded with MSCs for cartilage tissue repair of osteochondral defects. The composite scaffold was implanted in osteochondral defects for 12 weeks, and the cell-free scaffold was used as control. It was found that type II collagen staining was positive in cartilage tissue repaired by composite scaffold, indicating superior histological evaluation of hyaline chondrogenesis to that of cell-free scaffold. The MSCs combined with PEOT/PBT scaffold create a repair environment for cartilage repair. In cartilage tissue engineering using stem cells, it is important to stimulate proliferation and control stem cell differentiation into specific lineages. Zhu et al. [80] reported a gene-editing technique for articular cartilage repair, in which BMSCs transfected with connective tissue growth factor (CTGF) gene were combined with a PLGA scaffold. The animal model of the composite scaffold group successfully demonstrated hyaluroid cartilage regeneration similar to that of normal cartilage, and was superior to other groups in gross examination, qualitative and quantitative histological and mechanical evaluation. These findings suggest that CTGF modified BMSCs/PLGA scaffolds may be an alternative treatment for large osteocartilage defects at high-load sites. Similarly, Yang et al. [81] prepared a porous scaffold combined with C-type natriuretic peptide (CNP) gene modified BMSCs and CS/ SF to test its effect on the repair of whole-layer articular cartilage defects in rats. The results showed that CNP gene modified BMSCs and CS/ SF composite scaffold could lead to the formation of more cartilage matrix, and the repair effect was better than other groups. The above studies indicate that controlling MSCs to differentiate into chondrocytes through gene modification is expected to make a breakthrough in the repair of cartilage injury. However, it was also found that the addition of MSCs to autologous platelet-enriched fibrin scaffolds did not enhance cartilage repair [82]. It also indicates that the application of MSCs in promoting the repair of cartilage injury is particularly important to select appropriate biological scaffolds.

Other researchers are trying to use umbilical cord blood-derived MSCs (UCB-MSCs) to repair cartilage damage. Zheng et al. [83] investigated the effect of PCL-hydroxyapatite scaffolds loaded with UCB-MSCs on osteochondral regeneration. It was found that in rabbit osteochondral regeneration models using PCL-hydroxyapatite scaffolds, compared with the control group, PCL-hydroxyapatite scaffolds loaded with UCB-MSCs better promoted the repair of articular cartilage. Similarly, Huang et al. [84] discussed the effect of gelatin/hydroxyapatite composite scaffolds loaded with human umbilical cord blood-derived MSCs (hUCB-MSCs) in promoting cartilage repair. The scaffolds supported the adhesion, growth and proliferation of hUCB-MSCs and induced chondrocyte differentiation in vitro. The study also confirmed that hUCB-MSCs implantation in the injured site of pig articular cartilage can effectively repair cartilage defects. In addition, Chang et al. [85] investigated whether gelatin honeycomb scaffolds could enhance the proliferation, vitality and chondrogenic ability of human UCB-MSCs. Scaffold culture in cartilage differentiation medium was found to induce more stable expression of key genes COL2A1 and ACAN of hyaluronic cartilage, as well as production of type II collagen, agglutinoglycan, and glycosaminoglycan. In vivo studies also demonstrated that these UCB-MSCs differentiated chondrocytes stably expressed chondroid-related genes and proteins. These studies confirmed that biomaterial scaffolds can be used in cartilage injury repair by promoting UCB-MSCs survival and chondrogenic differentiation.

#### Adipose-derived stem cells (ADSCs)

ADSCs are promising for cartilage repair due to their easy access and chondrogenic potential. Kang et al. [86] discussed the feasibility of cartilage ECM scaffolds loaded with autologous ADSCs for the repair of rabbit cartilage defects. The results showed that all defects were completely filled with the repaired tissue using this scaffold, and most of the repaired sites were filled with hyaluronic cartilage 6 months after surgery. In order to better induce the differentiation of ADSCs into chondrocytes, Zheng et al. [87] prepared a composite scaffold through gene editing to control the chondrogenesis of ADSCs by transfecting the recombinant fusion protein LAP-MMP-mTGF-β3, thus effectively promoting the repair of cartilage damage. In this process, the addition of matrix metalloproteinases (MMPs) can trigger the release of recombinant fusion protein mTGF-β3 of LAP-MMP-mTGF-β3 in the combined scaffold, thus stimulating the differentiation of ADSCs into cartilage. The results of this study show that locally controlled delivery of LAP-MMP-mTGF-β3 constructs can accelerate the differentiation of ADSCs into cartilage in vivo, suggesting that this mixture has great potential for rapid treatment of OA. These studies indicate that ADSCs have great potential in cartilage injury repair, especially in inducing targeted differentiation by gene editing strategies.

#### Chondrocytes

The allogenic chondrocytes may be a seed cell source for cartilage tissue engineering. MAN Z et al. [88] studied the effect of CS hydrogel-demineralized bone matrix (DBM) mixed scaffold (CS/DBM) transplantation of allogenic chondrocytes in repairing rabbit cartilage injury. The results showed that several cartilage regeneration genes such as BMP-7, HGF, and IGF-1 were upregulated one month after transplantation. The study proved that the graft of allogeneic chondrocytes using CS/DBM scaffold is expected to successfully repair the rabbit cartilage injury, providing a new idea for cartilage tissue engineering. Similarly, Lin et al. [89] prepared a bilayer PLGA scaffold, trying to load chondrocytes to promote the regeneration of damaged cartilage. It was found that the double scaffold inoculated chondrocytes with tyramine treatment promoted osteocartilage regeneration and integration, thus achieving better articular cartilage repair. Furthermore, Ba et al. [90] compared the role of stromal vascular fraction cells (SVFs) and ADSCs co-cultured with chondrocytes in promoting cartilage regeneration by scaffolds composed of polyester material. The results showed that scaffolds implanted with SVFs and chondrocytes showed better in vivo healing outcomes. The above indicated that tissue engineering scaffolds loaded with chondrocytes have certain potential in cartilage injury repair.

### Biological scaffolds loaded with cytokines

It is well known that the differentiation of MSCs plays a decisive role in cartilage injury repair. Therefore, the researchers attempted to apply cytokines (TGF-β1, TGF-β3, MScs-specific affinity peptide, kartogenin) that can induce the differentiation of MSCs into cartilage onto biological scaffolds, in order to play a role in promoting cartilage repair (Fig. 2). Besides, the researchers also tried to load the scaffolds with platelet-rich plasma (PRP) rich in cytokines, which not only promote chondrogenesis, but also reduce local inflammatory response. The above conclusions have been confirmed in a large number of preclinical studies (Table 3).

**Table 3:** Preclinical study on the application of bioscaffolds loaded with cytokines in the repair of articular cartilage injury

Intervening measure	Joint site/implant site of cartilage injury models	Treatment outcomes	Animal model	References
A functional biomaterial using a biphasic scaffold platform and a BMSCs-specific affinity peptide	Trochlear groove of the femur	Functional biomaterials induced better cartilage repair without complications compared to conventional surgery or control scaffolds	Rabbits	Huang et al., [110]
MSCs E7 affinity peptide modified demineralized bone matrix (DBM) particles and CS hydrogel binding composite scaffold	Subcutaneous	This composite scaffold has the ability to promote translucency and superior chondroid structure formation in promoting chondrogenesis	Nude mice	Meng et al., [111]
Composite scaffold were formulated from a combination of HA fibers and PCL fibers, either with TGF-β3	Femoral trochlear groove	This composite scaffold improved histological scores and increased type 2 collagen content	Minipigs	Kim et al., [106]
Mechano growth factor (MGF) and TGF-β3 functionalized silk scaffold	Femoral trochlear groove	Fibroin scaffold loaded with TGF-β3 and MGF could enhance the recruitment of endogenous stem cells and promote the regeneration of articular cartilage in situ	Rabbits	Luo et al., [108]
Water-based polyurethane 3D-printed scaffold with controlled release function	Femoral trochlear groove	The scaffold promotes the self-aggregation of MSCs and induce the differentiation of MSCs into cartilage through timely release of bioactive components to generate substrates for cartilage repair	Rabbits	Hung et al., [123]
Functional scaffold named APM-E7 by conjugating a MSCs affinity peptide (E7) onto the acellular peritoneum matrix (APM)	Femoral trochlear groove	Functional scaffolds loaded with APM-E7 can provide space for MSCs and improve cell homing, two key factors required for cartilage tissue engineering	Rabbits	Meng et al., [112]
TGF-β1-releasing scaffold	Medial condyle of femur	The sustained release of TGF-β1 by this scaffold during osteochondral repair enhances early cartilage differentiation	Minipigs	Asen et al., [95]
Biomimetic cartilage scaffolds with orientated porous structure of two factors (kartogenin and TGF-β1)	Femoral trochlear groove	Biomimetic cartilage scaffolds can effectively repair cartilage defects, which is related to the scaffolds’ ability to guide the morphology, orientation, proliferation and differentiation of BMSCs	Rabbits	Wang et al., [97]
Sustained release SDF-1α/TGF-β1-loaded SF-porous gelatin scaffold	Femoral trochlear groove	This scaffold can promote homing, migration and chondrogenic differentiation of MSCs in vitro. In addition, SDF-1α and TGF-β1 have a synergistic effect in promoting chondrogenesis in vivo	Rats	Chen et al., [98]
Ginsenoside Rb1/TGF-β1 loaded biodegradable SF-gelatin porous scaffold	Femoral trochlear groove	The composite scaffold loaded with Rb1 and TGF-β1 can synergically create a microenvironment conducive to cartilage regeneration by promoting chondrogenesis and inhibiting inflammation levels in the body	Rats	Wu et al., [99]
Biofunctionalized chondrogenic shape-memory ternary scaffold	Femoral trochlear groove	Kartogenin has endowed this scaffold with biological activity that can better promote cartilage repair	Rats	Xuan et al., [114]
Nanofibrous HA scaffold delivering TGF-β3 and SDF-1α	Femoral trochlear groove	This scaffold loaded with SDF-1α and TGF-β3 can effectively promote cartilage formation	Minipigs	Martin et al., [109]
Biomimetic scaffold to deliver kartogenin for long-term cartilage regeneration	Femoral trochlear groove	HA composite lyotropic liquid crystal materials with joint protection and controlled drug release can be used as robust scaffolds to provide long-term cartilage repair	Rats	Wang et al., [116]
Solubilized articular cartilage ECM-derived scaffolds	The medial femoral condyle	This TGF-β3-loaded biological scaffold promoted advanced articular cartilage regeneration	Goats	Browe et al., [107]
A biomimetic scaffold using gelatin methacrylate (GELMA) and polyethylene glycol diacrylate (PEGDA) to wrap KGN	Femoral trochlear groove	The GELMA/PEDGA biomimetic scaffold modified by kartogenin repaired the cartilage defect and restored the cartilage to hyaline cartilage	Rabbits	Yu et al., [115]
Cell-free SF biomaterial scaffolds with bioactive molecules	Femoral trochlear groove	This SF-based biomimetic cartilage biofunctional scaffold with continuous controlled release of E7 and TGF-β1 may significantly promote cartilage regeneration in situ	Rabbits	Mao et al., [96]
A biomaterial scaffold with PRP containing SDF-1	Femoral trochlear groove	The PRF scaffold loaded with SDF-1 can better promote cartilage healing	Rabbits	Bahmanpour et al., [120]
Tricalcium phosphate scaffolds loaded with PRP	Distal articular surface of femur	The tricalcium phosphate scaffold loaded with PRP can well promote cartilage injury repair	Beagles	Li et al., [124]
Platelet-rich concentrates on a HA scaffold	Femoral trochlear groove	The combination of platelet concentrates rich in white blood cells with HA scaffold improves cartilage healing through various pathways	Bovine	Titan et al., [122]
Platelet lysate-rich plasma macroporous hydrogel (PLPMH) scaffold	Femoral trochlear groove	The PLPMH scaffold promote cartilage tissue regeneration by increasing the M2 macrophage ratio	Rabbits	Pan et al., [121]

HA: hyaluronic acid; PCL: poly ε-caprolactone; SF: silk fibroin; MSCs: marrow mesenchymal stem cells; BMSCs: bone marrow mesenchymal stem cells; ECM: extracellular matrix; CS: chitosan; SDF-1α: stromal derived factor-1α; PRP: platelet-rich plasma

#### TGF-β1

Continuous delivery of growth factors to the injured site is the key to creating a favorable microenvironment for cartilage repair. The persistence of transforming growth factor-β (TGF-β) is essential for inducing effective chondrogenesis. In order to study cartilage regeneration in cartilage defects, Asen et al. [95] explored the effect of TGF-β1 release scaffolds on improving cartilage repair in vivo. In vivo, sustained release of TGF-β1 increased the number of stromal derived factor-1 (SDF-1) positive cells in cartilage repair tissues, confirming that sustained release of TGF-β1 enhances early chondrogenic differentiation during osteochondral repair. Mao et al. [96] investigated the function of a cartilage biomimetic SF scaffold loaded with TGF-β1 for cartilage repair. In vivo studies showed that implantation of the biological functional scaffold significantly promoted in situ cartilage regeneration in rabbit cartilage defect models. This study also confirmed the importance of establishing a microenvironment that is low in inflammation and conducive to chondrogenic differentiation of endogenous stem cells for cartilage repair.

Researchers are also trying to use TGF-β1 in combination with other cytokines or drugs to improve the microenvironment for cartilage repair. Wang et al. [97] prepared a double-layer biomimetic cartilage scaffold loaded with kartogenin (KGN) and TGF-β1, whose surface layer was composed of collagen, CS and HA. A rabbit cartilage defect model was established and biomimetic cartilage scaffold was implanted in the defect area. The results showed that the biomimetic scaffold could effectively repair the defects, which was related to the scaffolds' ability to guide the morphology, orientation, proliferation and differentiation of BMSCs. Similarly, Chen et al. [98] constructed a novel SF-porous gelatin scaffold (SFPG) loaded with SDF-1/ TGF-β1. The SFPG scaffold consistently release SDF-1 and TGF-β1, which promote cartilage repair by promoting cell homing and chondrogenic differentiation. This SFPG scaffold was grafted to osteocartilage defects of knee joints in rats, and could promote cartilage regeneration and repair cartilage defects 12 weeks after transplantation. Studies have confirmed that this SFPG scaffold can promote homing, migration and chondrogenic differentiation of MSCs in vitro, and SDF-1 and TGF-β1 have a synergistic effect in promoting chondrogenesis in vivo. Furthermore, WU T et al. [99] designed a novel ginsenoside Rb1/TGF-β1 supported SF-gelatin porous scaffold, which has the function of reducing inflammation and promoting chondrogenesis. 12 weeks after surgery, the scaffold implantation in osteochondral defect of rats can effectively promote hyaluronic cartilage regeneration. The combination of Rb1 and TGF-β1 synergizes to create a microenvironment conducive to cartilage regeneration by promoting chondrogenesis and inhibiting inflammation levels in the body.

#### TGF-β3

In the process of cartilage repair and regeneration, TGF-β3 can effectively promote the differentiation of MSCs into chondrocytes and induce the expression of cartilage ECM by regulating the metabolism of articular chondroid-related multifunctional proteins in a time and concentration-dependent manner [100]. At the same time, studies have shown that TGF-β3 can inhibit the activity of inflammatory mediators such as IL-1 and TNF-α, while reducing the body's immune response [101, 102]. TGF-β3 plays a critical role in cartilage growth and reconstruction both in vitro and in vivo, which has been demonstrated by many previous studies [103, 104].

Kruger et al. [105] analyzed the potential of TGF-β3-loaded PGA scaffold to promote cartilage regeneration. The results showed that the cartilage differentiation ability of TGF-β3-PGA scaffold group was significantly better than that of control group. These results suggest that TGF-β3-PGA scaffold has chondrogenic potential. Similarly, Kim et al. [106] studied the ability of polymer scaffolds combined with TGF-β3 to improve cartilage repair. This composite scaffold is composed of HA and PCL fibers. At 12 weeks after implantation, the TGF-β3-loaded HA-PCL scaffold improved histological scores and increased type 2 collagen scaffold. Besides, Browe et al. [107] implanted TGF-β3-loaded chondrocyte ECM-derived scaffold into a goat cartilage defect model. In vivo, this TGF-β3-loaded scaffold promoted advanced articular cartilage regeneration. This study shows that TGF-β3 in combination with ECM-derived biomaterials has great potential for the treatment of articular cartilage defects.

The synergistic effect of TGF-β3 in conjunction with other cytokines in promoting cartilage repair was also investigated. Luo et al. [108] studied the synergistic effect of SF scaffolds loaded with mechanical growth factor (MGF) and TGF-β3 on promoting cartilage repair. These results indicate that the TGF-β3 and MGF functionalized SF scaffold can enhance endogenous stem cell recruitment and promote in situ articular cartilage regeneration, providing a new strategy for cartilage repair. Martin et al. [109] also developed an electrospun cell-free fibrous HA scaffold loaded with SDF-1 and TGF-β3. The results showed that SDF-1 and TGF-β3 could promote the migration of MSCs, and TGF could also promote the matrix formation of MSCs. These results suggest that the search for synergistic growth factor and TGF-β3 combination is particularly important in promoting cartilage repair.

#### Specific affinity peptide of MSCs

MSCs are known to play an important role in cartilage repair. Huang et al. [110] constructed a CS biphasic scaffold loaded with BMSCs specific affinity peptides. The biomaterial can stimulate stem cells proliferation and cartilage differentiation during in vitro culture. In vivo studies have demonstrated that this functional biomaterial can better induce cartilage repair without complications. Similarly, Meng et al. [111] constructed a composite scaffold combining MSCs E7 affinity peptide modified demineralized bone matrix (DBM) particles and CS hydrogels. In vitro studies have shown that the DBM-E7/CS scaffold significantly promote the survival of rat BMSCs than CS or DBM/CS scaffolds. At the same time, DBM-E7/CS scaffold increased the matrix production of BMSCs and improved the ability of cartilage differentiation of BMSCs. Similarly, in vivo studies have demonstrated superior chondroid structure generation after treatment with the DBM-E7/CS scaffold. Additionally, Meng et al. [112] established a functional scaffold named APM-E7 by coupling a MSCs affinity peptide E7 to the acellular peritoneal matrix (APM). In vitro culture, APM-E7 scaffold could better support proliferation of BMSCs and better differentiation into chondrocytes. 24 weeks after implantation of APM-E7 scaffold, rabbit cartilage defects showed better quality of new cartilage and no graft rejection. These studies suggest that MSCs-specific affinity peptides have potential value in cartilage repair due to their ability to promote MSCs proliferation and cartilage differentiation.

#### Kartogenin

KGN was first reported in 2012 to induce MSCs to differentiate into chondrogenic chondrocytes by stimulating RUNX1 expression, showing great potential to promote chondrogenesis [113]. KGN combined with biological scaffolds has been widely used in the study of promoting cartilage repair and has shown promising potential in promoting cartilage repair [114–116]. Xuan et al. [114] prepared the polyglycerin sebacate (PGS)/ polypropyl sebacate (PPS) composite scaffold, and the bioactive KGN endows the scaffold with chondrogenic ability. The results showed that the PPS/PGS/KGN scaffold promoted chondrogenic differentiation of BMSCs and inhibited osteogenic differentiation in a concentration-dependent manner, and effectively promoted cartilage regeneration in the full-layer defect of femoral patellar sulcus in rats. Similarly, Yu et al. [115] prepared a biomimetic scaffold using gelatin methacrylate (GELMA) and polyethylene glycol diacrylate (PEGDA) to wrap KGN in a biomimetic scaffold. With the degradation of this scaffold, scaffold loaded KGN is slowly released, inducing endogenous MSCs to differentiate into chondrocytes to repair damaged cartilage tissue. This study confirmed that GELMA/PEGDA-KGN has the function of repairing cartilage defects.

#### Platelet-rich plasma

PRP is a platelet concentrate obtained by centrifugation of whole blood. It has a higher platelet concentration than normal whole blood and contains a variety of growth factors. PRP has been widely used in orthopedic injury-related diseases by secreting a large number of cytokines, chemokines and growth factors, reducing the occurrence of inflammation, promoting angiogenesis, promoting the proliferation and differentiation of chondrocytes, and thus promoting the healing of osteochondral injury [117, 118]. Sermer et al. [119] studied the potential of PRP-supported scaffolds or bioengineering implants in enhancing the repair of articular cartilage through literature search. The study reviewed 14 animal model studies, 10 of which reported a positive effect of PRP, while only two showed an overall negative effect. The remaining 2 studies reported no significant difference or neutral results with PRP. This study indicates that PRP combined with biological scaffolds still has great potential in articular cartilage repair, and further optimization of the structure and materials of their composite scaffolds is needed.

Bahmanpour et al. [120] constructed a scaffold with PRP containing SDF-1 to induce hyaluronic cartilage regeneration in deficient rabbit knee joints. ICRS, microscopic analysis and type II collagen immunofluorescence staining showed that the score of PRP-SDF-1 group was higher than that of other groups. This study suggests that PRP with high levels of cytokines combined with biomaterial scaffold has great potential in cartilage repair. Similarly, Pan et al. [121] developed a macroporous hydrogel scaffold rich in platelet lyase plasma for continuous recruitment and polarization of endogenous anti-inflammatory M2 macrophages to improve the repair of cartilage defects. This study found that this scaffold could increase the proportion of M2 macrophages and promote cartilage tissue regeneration in a rabbit cartilage defect model. These studies suggest that the scaffold has great potential in articular cartilage tissue engineering by providing an anti-inflammatory and pro-regenerative microenvironment. Furthermore, Titan et al. [122] discussed the role of HA scaffold loaded with human leukocyte-rich platelet plasma (L-PRP) in promoting the repair of cartilage defects. This study demonstrated that the combination of L-PRP concentrates with HA scaffold can improve cartilage healing through various pathways. These studies indicate that biological scaffolds loaded with PRP have great potential in promoting the healing of cartilage injury.

### Hydrogel

Functional tissue engineering provides a new approach for the repair and regeneration of damaged cartilage. Hydrogels are widely used because of their advantages such as rapid filling of defects, proper structural support, cell aggregation and biocompatibility of matrix deposition. Cartilage tissue engineering offers a promising regeneration strategy, and the use of injectable hydrogels as scaffolds has recently attracted a lot of attention. Researchers have conducted a large number of preclinical studies on the application of hydrogels from different sources in osteochondral injury repair in different ways (Table 4), and most of the research results reflect the superiority of hydrogels in promoting cartilage injury repair. Hydrogels are widely used as cells/growth factors carriers for tissue engineering scaffolds to promote cartilage repair and regeneration or as permanent implants to replace damaged cartilage (Fig. 2). Hydrogels have the unique advantages of repairing large tissue defects, avoiding donor complications and two-stage invasive surgery, and have shown better cartilage repair results [125].

**Table 4:** Preclinical study of hydrogels applied to repair articular cartilage injury

Intervening measure	Joint site/implant site of cartilage injury models	Treatment outcomes	Animal model	References
Bilayered, biodegradable hydrogel composites loaded with dual growth factors	Medial condyle of femur	Hydrogel composites loaded with insulin-like growth factor-1 (IGF-1) and bone morphogenetic protein-2 (BMP-2) have shown potential as space-guided multiple growth factor release vectors for osteochondral tissue repair	Rabbits	Lu et al., [137]
Injectable and thermosensitive TGF-β1-loaded poly(ε-caprolactone)-poly (ethylene glycol)-poly(ε-caprolactone) (PCEC) hydrogel system	The femoral trochlear groove	The cell-free hydrogel has the ability to repair cartilage in vivo	Rats	Zhou et al., [131]
A SF and carboxymethyl chitosan composite hydrogel	Subcutaneous	This composite hydrogel has adjustable material properties, degradation rate and good biocompatibility. It is a promising scaffold material for cartilage tissue engineering	Mice	Li et al., [129]
A visible light-activatable methacrylated gelatin hydrogel with methacrylated HA	The femoral trochlear groove	After 12 weeks of implantation, the composite hydrogel can promote the regeneration of cartilage and subchondral bone, and can be used for the repair and resurfacing of articular cartilage defects	Rabbits	Lin et al., [126]
Methacrylate gelatin hydrogel loaded with dasatinib	The femoral trochlear groove	The hydrogel loaded with dasatinib can promote cartilage repair by promote chondrogenic differentiation of MSCs	Rabbits	Nie et al., [138]
Autologous PRP combined with injectable HA hydrogel	Medial condyle of femur	Autologous PRP combined with HA hydrogel has the ability to promote cartilage healing and regeneration in osteochondral defects	Porcine model	Yan et al., [133]
An injectable hydrogel crosslinked by strontium-doped bioglass	Femoral trochlear groove	In cartilage repair, the composite hydrogel promotes the close binding of the original tissue and the formation of new chondroid tissue	Rats	Cai et al., [130]
The combination with 3D-printed rigid PLGA scaffold with cell-laden PRP hydrogel	Femoral trochlear groove	The composite hydrogel not only has good cell transport and growth factor release ability, but also has good mechanical strength, which can be applied to the regeneration of bone cartilage	Rabbits	Tang et al., [134]
An injectable hydrogel using SF in a novel one-step ultrasonication crosslinking method	Femoral trochlear groove	This hydrogel has good physical, chemical and biomechanical properties, it can promote cartilage regeneration	Rabbits	Yuan et al., [128]
Hydrogel scaffold linked with bioactive peptides that can selectively adsorb TGF-β1	Femoral trochlear groove	This study showed that porous hydrogels combined with peptides can promote cartilage tissue regeneration	Rats	Ding et al., [132]
BMSCs affinity peptide sequence PFSSTKT (PFS)-modified chondrocyte ECM particles combined with GelMA hydrogel	Femoral trochlear groove	The GelMA/ECM-PFS functional hydrogel system promoted the recruitment of endogenous MSCs from the defect site, thus effectively promoting hyaluronic cartilage formation	Rabbits	Huang et al., [135]
Photo-annealed granular hydrogel composed of HA, polyethylene glycol, and gelatin	Femoral trochlear groove	The hydrogel can significantly stimulate hyaluronic cartilage matrix deposition and articulation, thus promoting hyaluronic cartilage regeneration	Rats	Zhu et al., [127]
3D scaffolds of acellular cartilage ECM loaded with MSCs-derived exosomes (MSC-Exos)	Femoral trochlear groove	3D scaffolds loaded with MSC-Exos demonstrated superior cartilage repair ability	Rats	Yan et al., [136]

HA: hyaluronic acid; MSCs: marrow mesenchymal stem cells; BMSCs: bone marrow mesenchymal stem cells; ECM: extracellular matrix; PRP: platelet-rich plasma; PLGA: polylactic acid-glycolic acid

Lin et al. [126] further optimized methacrylate gelatin (mGL) hydrogel by adding methacrylate HA (mHA) and discussed its ability to promote cartilage repair. A cartilage defect was established on the condyle of rabbit femur, and this scaffold was used to fill the lesion. The results show that mGL/mHA composite scaffold can promote cartilage and subchondral bone regeneration after implantation, and support its application as a promising scaffold for the repair of articular cartilage defects. Recently, Zhu et al. [127] formulated a hydrogel composed of HA, polyethylene glycol and gelatin for cartilage regeneration. In vitro studies have shown that the hydrogel can promote the volume expansion and morphological recovery of chondrocytes, and significantly improve the chondroblast phenotype. Further in vivo studies showed that the hydrogel loaded with chondrocytes significantly stimulated hyaline cartilage matrix deposition and connection, thereby promoting hyaline cartilage regeneration. This study demonstrated the feasibility of the hydrogel loaded with chondrocytes as an in situ chondrocyte deployment scaffold for cartilage regeneration, providing new ideas for the design of hydrogel scaffolds for cartilage tissue engineering.

SF is an advanced natural material that can be used to construct non-toxic injectable hydrogels that can be effectively used in crosslinking applications. Yuan et al. [128] developed an injectable ultrasonication-induced SF (US-SF) hydrogel and systematically evaluated the gel kinetics and properties of ultrasonication-induced SF hydrogel. The results showed that the new ultrasonic induced SF hydrogel had good physical, chemical and biomechanical properties. It has been demonstrated in vitro and in vivo to promote cartilage regeneration, suggesting that it may be a potential solution for cartilage repair and regeneration. Furthermore, Li et al. [129] developed a composite hydrogel of SF and carboxymethyl chitosan (CMCS) with enzyme crosslinking. In vitro cell experiments promoting cartilage repair showed that the hydrogel supported adhesion, proliferation, glycosaminoglycan synthesis and chondroblast phenotype of rabbit articular chondrocytes. Finally, the hydrogel implanted under the skin of mice did not show infection or local inflammation, and it has good biocompatibility in vivo. These studies suggest that SF hydrogels are a promising strategy for cartilage tissue engineering.

BMSCs can differentiate into chondrocytes, which play an important role in cartilage repair. Additionally, uncontrolled inflammatory responses to implants can compromise scaffold stability and cartilage regeneration outcomes. Cai et al. [130] prepared an injectable hydrogel to regulate the differentiation and inflammatory response of human BMSCs (hBMSCs) by adding strontium bioglass (SrBG) crosslinking. In vitro studies have shown that the SA/SrBG scaffold can induce the differentiation of hBMSCs into chondrocytes by promoting the polarization of macrophages towards M2 phenotype. The new chondroid tissue with smooth surface and close adhesion to the original tissue can be found by injecting the composite hydrogel into the cartilage defect model. This study shows that synergistic strategies based on enhanced differentiation ability and regulation of inflammatory responses are promising and may lead the way to novel anti-inflammatory biomaterials.

TGF-β1 is thought to promote chondrogenesis. Zhou et al. [131] prepared a new type of TGF-β1-loaded PCL hydrogel. The study confirmed that the material has good porous structure, good injectable properties and sustained drug release. In vivo studies further demonstrated the ability of the new hydrogel to promote cartilage regeneration. To optimize the structure of the hydrogel, Ding et al. [132] recently constructed a double-layer porous scaffold using gelatin-methylacryloyl (GelMA) hydrogel as the substrate. The upper layer was covalently bonded with bioactive peptide adsorbing TGF-β1 for cartilage repair, and the lower layer was blended with hydroxyapatite for subchondral regeneration. The results of cartilage and osteogenesis induction in vitro and osteochondral repair in vivo showed that the scaffolds had good therapeutic effect.

PRP is rich in a variety of growth factors, proteins and cytokines, which can promote cartilage healing by stimulating cell proliferation and inducing chondrogenesis at the site of cartilage defect. Yan et al. [133] discussed the feasibility of autologous PRP combined with injectable HA hydrogel in the treatment of cartilage injury. It was found that the hydrogel had the ability to promote cartilage regeneration in the osteochondral defect of pig femur. Besides, Tang et al. [134] combined the advantages of a 3D-printed rigid PLGA scaffold with cell-loaded PRP hydrogel. In this study, PRP hydrogels achieved effective delivery of MSCs to PLGA scaffolds. The results show that this unique hybrid system with good cell transport ability, growth factor release ability and good mechanical strength can be practically applied to the regeneration of osteochondrome, which will greatly promote the development of cartilage tissue engineering.

Huang et al. [135] constructed chondrocyte ECM granules modified with affinity peptide sequence of BMSCs to bind GelMA hydrogel. In vitro experiments showed that the solid-supported composite scaffold had appropriate pore size and porosity, and the scaffold provided a 3D microenvironment that supported cell adhesion, proliferation, and chondrogenic differentiation. In vivo experiments also showed that the GelMA/ECM could recruit rabbit BMSCs to migrate to the cartilage injury site, and then successfully promote cartilage formation. Improving the poor microenvironment in the joint cavity has the potential to treat cartilage damage, and MSCs-derived exosomes (MSCs-Exos), which can regulate cell behavior, are emerging as a new cell-free cartilage repair therapy. YAN Z et al. [136] studied whether MSCs-Exos cultured on 3D scaffolds could improve the poor joint cavity microenvironment caused by cartilage injury, and discussed its mechanism. In vitro experiments have shown improved efficiency of MSCs-Exos cultured on 3D scaffolds (3D-Exos). In vivo studies found that 3D-Exos showed strong cartilage repair ability in the knee osteochondral defect model of rats after the micropore adsorption of scaffolds. In addition, researchers also investigated the role of insulin-like growth factor-1 (IGF-1), bone morphogenetic protein-2 (BMP-2), other cytokines [137], dasatinib [138] and other drugs in promoting the repair of cartilage injury. Studies have confirmed that hydrogels loaded with cytokines or drugs have certain efficacy in cartilage repair.

## Clinical study of tissue engineering strategies applied to repair articular cartilage injury

In recent years, biological scaffold technology has been gradually applied in the clinical treatment of cartilage injury repair, and researchers have also conducted some clinical studies (Table 5). Most of the studies obtained good clinical efficacy, some studies did not obtain positive results. Biological scaffolds used in cartilage repair come from a wide range of sources, and the scaffolds used vary greatly in structure and properties, leading to different therapeutic effects.

**Table 5:** Clinical study of tissue engineering strategies in the repair of articular cartilage injury

Intervening measure	Research type	Studies/patients(n)	Treatment outcomes	Disease type	References
A cell-free collagen-hydroxyapatite osteochondral scaffold	Case series	27 patients	This study highlights the safety and potential of the scaffold for the treatment of osteochondral knee defects with good clinical results and stable interim follow-up results	Cartilaginous lesions of the knee joint	Kon et al. [139]
The MaioRegen(®) scaffold is a cell-free biomimetic scaffold consisting of type I collagen and hydroxyapatite	Prospective therapeutic study	10 patients	Treatment of osteochondral defects with biomimetic scaffolds resulted in incomplete cartilage repair and poor subchondral bone repair at 2.5 years of follow-up	Cartilaginous lesions of the knee and talus bones	Christensen et al., [145]
Cell-free osteochondral scaffold	Case series	27 patients	Cell-free osteochondral scaffold implantation in the treatment of osteochondritis dissecans has shown good and stable results	Knee osteochondritis dissecans	Perdisa et al., [140]
A cell-free collagen type I scaffold	Case series	28 patients	The application of this scaffold for large defects showed increased wear of the repair tissue and clinical failure in 18% of cases at 5-year follow-up	Cartilage defects of the knee	Schüttler et al., [146]
Scaffolds with hyaluronan and CS-based structure	retrospective comparative study	81 patients	Both scaffolds are effective for cartilage healing, but neither has clinical or radiological advantage	Talus osteochondral lesions	Akmeşe et al., [147]
A cell-free aragonite-based scaffold	Case series	13 patients	This scaffold is a safe and clinically effective implant for the treatment of small and medium-sized joint surface lesion in the distal femur. The implants showed satisfactory bone integration and osteochondral recovery for up to 3 years	Joint surface lesion of the knee	Van Genechten et al., [150]
The Igor scaffold	Case series	21 patients	Patients using Igor scaffold reported reduced pain and increased activity, with most reporting good results	Locally restricted cartilage defects	Zak et al., [143]
Two scaffolds with hyaluronan-based and CS-based structure	Retrospective comparative study	69 patients	Both scaffolds are useful for cartilage regeneration, but have no clinical or radiological advantage	Symptomatic condylar osteochondral lesions	Akmeşe et al., [148]
Collagen scaffold	Retrospective cohort study	94 patients	The use of collagen scaffold may not affect clinical efficacy or imaging results	Osteochondral lesions of the talus	Gorgun et al., [149]
Scaffold-supported bone marrow-derived cells	Therapeutic case series	64 patients	The scaffold was used to repair osteochondral lesions of the talus, and the clinical effect was significant and lasted for a long time	Osteochondral lesions of the talus	Buda et al., [141]
The 3D scaffold based on a bilayered collagen type I sponge	Case series	23 patients	The use of this scaffold is a feasible method for the treatment of large focal osteochondral defects, and the recent clinical and imaging results are good	Cartilage damage and OA of the knee	Zak et al., [151]
MSCs-loaded fibrin glue scaffold	Retrospective cohort study	54 patients	The International Cartilage Repair Society (ICRS) score was higher after the treatment of knee joint with fibrin glue loaded with MSCs	Osteoarthritic (OA) knees	Kim et al., [142]
Implant of hydrogel	Systematic review and meta-analysis	50 studies/ 2846 patients	There were clinically and statistically significant improvements in pain scores (VAS and WOMAC) and functional scores (IKDC and Lysholm) after treatment of knee cartilage injury with hydrogels compared to before treatment	Knee joint cartilage regeneration	Hosseini et al., [144]

MSCs: marrow mesenchymal stem cells; CS: chitosan

Kon et al. [139] evaluated the medium-term clinical effect of the cytofree collagen-hydroxyapatite osteocartilage scaffold and recorded the imaging evolution of tissue regeneration during 5-year follow-up by magnetic resonance imaging (MRI) analysis. The study included 27 patients who were followed for two to five years and clinically assessed using the International Knee Literature Committee (IKDC) and tegner scores. Results of the study found statistically significant improvements in all clinical scores from initial assessment to 2 to 5 years of follow-up. The osteochondral scaffold can be used for the single step treatment of cartilage and osteochondral knee defects. The results underscore the safety and potential of the procedure, providing good clinical outcomes and stable interim follow-up results. Similarly, Perdisa et al. [140] evaluated the mid-term clinical and imaging effects of acellular osteochondral scaffold implantation in the treatment of knee obsessive–compulsive disorder (OCD). Twenty-seven patients treated for OCD of the knee were prospectively evaluated for up to 5 years. All patients significantly improved their clinical scores at each follow-up up to the final assessment. The study demonstrated good and stable outcomes for the treatment of obsessive–compulsive knee disorders with this cell-free scaffold implantation over a follow-up period of up to 60 months. MRI showed abnormalities, particularly at the subchondral bone level, but overall features improved over time.

To investigate the ability of bone marrow-derived cells loaded scaffolds for cartilage repair, Buda et al. [141] developed a biological scaffold for cartilage repair using a HA scaffold loaded with bone marrow-derived cells and a platelet gel. The ability of this technique to repair 64 talus cartilage lesions was investigated in a prospective clinical study. Scaffolds loaded with bone marrow-derived cells were used to repair osteochondropathy of the talus with remarkable clinical effect and prolonged duration. Besides, Kim et al. [142] reported the clinical efficacy and arthroscopic follow-up results of fibrin glue loaded with MSCs as a scaffold for knee patients with OA, and compared it with MSCs without scaffold implantation. In this study, 54 patients with osteoarthritic knee cartilage lesions underwent arthroscopic review after implantation of BMSCs. The efficacy of cartilage repair was evaluated according to the ICRS scores. Follow-up results showed that the treatment group loaded with MSCs scaffolds had higher ICRS scores. These studies provide clinical evidence that biological scaffolds loaded with MSCs have potential value in the treatment of cartilage injury.

For large local cartilage defects in young patients, various scaffolds loaded with autologous chondrocytes have shown good medium and long-term results. Zak et al. [143] evaluated the clinical and radiological outcomes at 2-year follow-up in patients undergoing autologous chondrocyte transplantation (ACI) in the knee joint using the Igor scaffold (Igor Tissue Organ Reconstruction Institute). A total of 21 patients with Igor scaffold combined with ACI were studied. The majority of patients were found to have good to excellent clinical and radiological scores during follow-up. 90.5% of cartilage defects were satisfied with filling and fusion. This study also showed that the clinical and radiological outcomes of third-generation ACI using Igor scaffolds were comparable to those of other scaffolds in patients with large traumatic or degenerative cartilage defects. Furthermore, a large number of clinical studies have been conducted on hydrogel scaffolds in improving cartilage repair. Hosseini et al. [144] explored the effectiveness of hydrogel treatment for defects of knee cartilage (femoral condyle, patella, tibial plateau and trochlea) through systematic review and meta-analysis. Fifty clinical trials were retrieved, including 2846 patients, of whom 986 received cell-free hydrogels and 1860 used cell-free hydrogels. This meta-analysis demonstrated clinically and statistically significant improvements in pain scores (VAS and WOMAC) and functional scores (IKDC and Lysholm) after the use of hydrogel compared with before treatment. Thus, the current evidence shows the effectiveness of hydrogel therapy in correcting and repairing cartilage defects in the knee.

However, some studies have confirmed that biological scaffolds have no obvious clinical effect in promoting the repair of cartilage injury, and some studies even found that some biological scaffolds may cause poor cartilage repair [145–149]. MaioRegen(^®^) is a cell-free biomimetic scaffold composed of type I collagen and hydroxyapatite. CHRISTENSEN B B et al. [145] evaluated the clinical efficacy of MaioRegen(^®^) scaffold for osteocartilage repair. At 1 and 2.5 years of follow-up, the use of this biomimetic scaffold for osteochondral defects in the ankle and knee resulted in incomplete cartilage repair and poor subchondral bone repair. Similarly, Schuttler et al. [146] reported interim clinical, radiological, and histological results of cartilage repair with cell-free collagen I scaffolds. The use of this cell-free type I collagen scaffold to repair large defects resulted in increased wear and tear on the repaired tissue and clinical failure in 18% of cases during the 5-year follow-up. Additionally, Akme et al. [148] compared the clinical and radiological outcomes of two different HA or CS-based scaffolds for the treatment of symptomatic condylar osteochondropathy. The study included 69 patients who underwent HA or CS scaffold surgery to repair osteocartilage damage. The results show that both scaffolds are useful for cartilage regeneration, but have no clinical or radiological advantage. These studies indicate that biological scaffolds need to be better optimized to meet the needs of clinical transformation.

## Dilemmas of tissue engineering strategies applied to the repair of articular cartilage injury

Tissue engineering scaffolds used in cartilage repair should have two basic elements: biocompatibility and the ability to promote the growth of chondrocytes. Good tissue scaffolds, cytokines, or seed cells that promote the proliferation and differentiation of chondrocytes are the preconditions for successful repair of damaged cartilage by tissue engineered biological scaffolds. However, in clinical work, it is difficult to find suitable cartilage tissue replacements, resulting in the slow development of tissue engineering technology for osteochondral repair. In recent years, with the development and innovation of materials science and cell biology, bone tissue engineering technology has brought new hope for the regeneration and repair of cartilage injuries.

As mentioned above, the structural properties of osteochondral tissue are extremely complex, and cartilage tissue regeneration remains a great challenge for tissue engineering. Among osteochondral tissues, articular cartilage and subchondral bone are tissues with a variety of microstructures. Therefore, in order to promote and enhance osteochondral regeneration, the ideal bioactive scaffold should have the natural structure and physiological characteristics similar to articular cartilage and subchondral bone tissue. Biphasic and multilayered scaffolds were prepared to simulate the layered structure of osteochondral tissue [40, 66, 89, 97]. However, the biological simulation of the original structure of osteochondral tissue remains difficult, and the clinical application of biphasic and multilayer scaffolds is limited by uneven mechanical response and low adhesion strength between adjacent layers. At present, the methods of preparing bioactive scaffolds mainly include phase separation, gas foaming, freeze drying, and space fixer. The main function of the traditional method is to adjust and control the microstructure and mechanical properties of the scaffolds. In recent years, the emergence of 3D printing technology has provided a feasible strategy for the preparation of layered structures of biological scaffolds used in the regeneration of osteochondral tissue [42, 49, 136].

The cartilage layer mainly uses polymers and ECM, while the subchondral bone layer mostly uses metal materials, bioceramics, and other materials. In order to improve the specific biological function of osteochondral regeneration, the researchers loaded growth factors and bioactive substances into the scaffold material. Furthermore, specific cells including stem cells and chondrocytes were implanted on the scaffold to improve the efficiency of cartilage tissue regeneration. There are also studies using gene editing to alter the genetic structure of stem cells so that they differentiate in the direction of chondrocytes [80, 81]. In the process of commercialization, scaffold materials containing growth factors and cells are difficult to store and transport due to low cell survival rate and instability of growth factors. Most commercial scaffolds are cell and growth factor free. At the same time, the large-scale preparation of bioactive factors and the intervention of gene editing means of stem cells will lead to high medical costs, which is also the problem faced by the clinical application of tissue engineering scaffolds.

Furthermore, hydrogels, as carriers of cells, growth factors, and other regulatory substances, have been used as tissue engineering scaffolds to repair damaged cartilage, and have shown good effects in a large number of in vivo and in vitro experiments [126–131]. Hydrogels prepared from natural biomacromolecules and their derivatives have good biocompatibility and biodegradability, but their mechanical strength is often weak, which limits their application as load-bearing support materials. Synthetic polymer hydrogels can accurately regulate mechanical properties, but cell affinity and biodegradability are usually poor. By combining the advantages of two different types of hydrogels, natural and synthetic polymers, a hybrid hydrogels can be prepared to offer both of these advantages. However, there are some obstacles to the clinical transformation of hydrogels with complex structure. This is because the preparation of composite hydrogels using advanced biomanufacturing techniques usually requires special formulations and equipment, and its preparation time is relatively long and the cost is much higher than that of simple structured hydrogels products. At the same time, the influence of different components, morphological structures and physical and chemical properties of hydrogels on cartilage repair is not clear, and there are many confounding factors, which undoubtedly increases the difficulty of clinical transformation of hydrogels with complex structures.

MSCs from different sources have shown potential to repair cartilage defects by differentiating into chondrocytes. The combined use of MSCs, growth factors and biological scaffolds provides a new model for cartilage repair, and a large number of studies have confirmed its efficacy [83–85, 111, 112]. Under the action of growth factors, the transplanted MSCs can be qualitatively differentiated into chondrocytes, which can form a suitable mechanical strength interface between the new cartilage and the surrounding cartilage tissue to achieve complete union. However, the survival of transplanted cells and the fusion of cells with host tissues are of great concern. The actual potential of this technique in cartilage repair needs further investigation. Besides, appropriate cell sources should be investigated to determine the source of autologous or allogenic cells that can be used for MSCs. Although scaffold characteristics clearly influence chondrogenesis, the exact mechanism that promotes chondrogenesis remains to be elucidated.

At present, the functional studies of tissue engineering scaffolds used in cartilage injury repair are mostly limited to preclinical studies. In order to enhance the function of biological scaffolds in cartilage injury repair, researchers tend to integrate various technologies in the design of scaffold structure. However, the final product is not conducive to clinical transformation due to its complex structure. Therefore, it is necessary to further study the natural physiological process of cartilage repair in the future, and prepare a simple and effective biological scaffold to bring hope to clinical patients. Furthermore, most of the relevant clinical studies are case reports and retrospective single-arm clinical studies with a low level of evidence recently. In order to promote the widespread application of tissue engineering scaffolds in clinical practice, prospective randomized controlled clinical studies are needed to evaluate the true function of tissue engineering scaffolds in repairing cartilage injury.

## Conclusions

In summary, due to the poor self-healing ability of articular cartilage, researchers try to apply tissue engineering materials to cartilage injury repair. In recent years, tissue engineering scaffolds have been gradually applied to the study of cartilage injury repair. A large number of preclinical and clinical studies have also confirmed its potential in repairing cartilage damage. However, the optimization process of tissue engineering scaffolds inevitably brings economic burden and histocompatibility problems. In addition, most existing clinical studies are case series reports and retrospective studies with low levels of evidence. Therefore, in the future, it is necessary not only to optimize the histocompatibility and safety of materials but also to improve the feasibility of clinical transformation. More prospective randomized controlled clinical studies are needed to confirm its ability to promote cartilage repair.

## Data Availability

The literatures summarized in this study were all from PUBMED database, and the figures used were all made by the team themselves.
